# A New Languidulane Diterpenoid from *Salvia mexicana* var. *mexicana*

**DOI:** 10.3390/molecules16108866

**Published:** 2011-10-21

**Authors:** Bernardo Antonio Frontana-Uribe, Martha Verónica Escárcega-Bobadilla, Rosa Estrada-Reyes, José Antonio Morales-Serna, Manuel Salmón, Jorge Cárdenas

**Affiliations:** Instituto de Química, Universidad Nacional Autónoma de México, Circuito Exterior, Ciudad Universitaria, 04510 México D.F., México; Email: bafrontu@unam.mx (B.A.F.-U.); mescarcega@iciq.es (M.V.E.-B.); restrada@imp.edu.mx (R.E.-R.); morser@unam.mx (J.A.M.-S.); salmon@unam.mx (M.S.)

**Keywords:** *Salvia mexicana*, Labiatae, diterpene, languidulanes, NMR

## Abstract

From the aerial parts of *Salvia mexicana* var. *mexicana*, two C-10 epimers (α and β) of salvimexicanolide were isolated. Our interpretation of the data, especially the ^13^C NMR, led us to conclude that the previously described ^13^C-NMR spectrum of the α-epimer was not accurately assigned and it actually corresponds to the β-epimer. The structures proposed for the salvimexicanolides were verified by means of NOESY experiments. Dugesin B, arbutin, naringenin and the mixture of oleanolic and ursolic acids were also isolated from this *Salvia* spp.

## 1. Introduction

*Salvia* [1] is one of the largest genera of plants, with over 500 species distributed throughout Mexico, Central and South America. In Mexico, it is represented by over 300 species, many of which are endemic [2]. The diterpenoid constituents of genus *Salvia* are closely related to the subgenus and section to which they belong. After an intensive study of Mexican *Salvia* species, a great number of *neo*-clerodane diterpenoids have been isolated [3,4]. Some examples of the languidulane skeleton, which could be biogenetically originated from the junction of the C-16 and C-1 of a clerodane skeleton [5], have been found in this genus [6].

*Salvia Mexicana* (Subgenus Calosphace), comprises the varieties *major*, *minor* and *mexicana* [1,2]; which are endemic to the central states of Mexico. In 1999 Esquivel and co-workers reported the results of their study of *Salvia mexicana* var. *major*, to our knowledge the only variety that has been investigated. In that study, a new languidulane diterpenoid was described [7]. In this context, this paper describes the isolation and structural elucidation of a new languidulane diterpene, named salvimexicanolide B (**2**), from the aerial part of *Salvia mexicana* var. *mexicana*. In addition to the above mentioned compound, ursolic and oleanolic acids, salvimexicanolide (**1**), dugesin B (**3**), the flavanone (-) naringenin (**4**) and the substituted sugar arbutin (**5**) were identified. 

## 2. Results and Discussion

**Figure 1:**
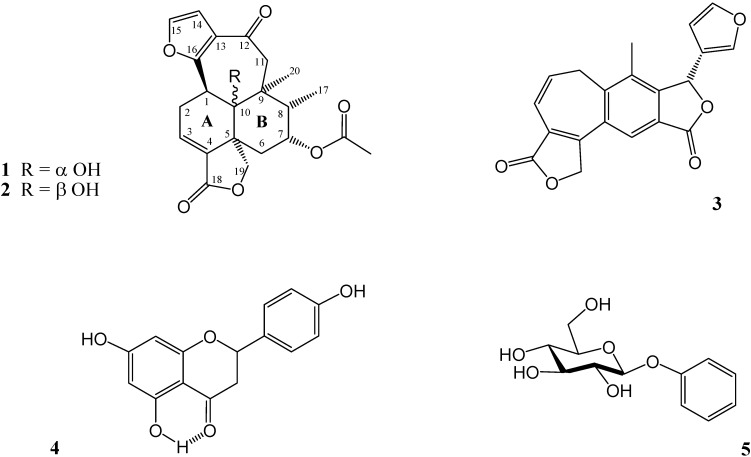
Products isolated from *S. mexicana* var. *mexicana*.

The aerial part of *Salvia mexicana* var. *mexicana* was dried, ground and extracted with acetone at room temperature in a closed container for one week, as described in the Experimental. After extensive column chromatography, six known natural products were isolated and identified. These were ursolic acid, oleanolic acid [8], salvimexicanolide (**1**) [7], dugesin B (**3**) [9,10], naringenin (**4**) [11] and arbutin (**5**) [12]. Their structures (Figure 1) were confirmed by comparing their spectra with authentic ones. To our delight, were also able to isolate a new compound from the same extract, salvimexicanolide B (**2**), which is structurally related with salvimexicanolide (**1**).

Salvimexicanolide B (**2**) is a yellowish crystalline product (mp. 137–139 °C) whose MS was consistent with a molecular formula of C_22_H_24_O_7_, the molecular formula of **1**. The IR spectrum shows the presence of γ-lactone (1777 cm^−1^), hydroxyl (3604 cm^−1^), an ester carbonyl (1738 cm^−1^) and unsaturated ketone carbonyl (1660 cm^−1^) groups. The hydroxyl group observed in the IR spectrum matches a broad signal exchangeable with D_2_O in the ^1^H-NMR spectrum (δ 3.38) and was confirmed by means of a ^13^C-NMR spectrum (δ 77.8). The ^1^H-NMR spectrum obtained for this product (Table 1) shows signals similar to those described for the disubstituted furan ring, and the ABCX system, assigned to H-1, H-2_eq_, H-2_ax_ and H-3 in the α-epimer. However, some other signals are displaced or present a different shape. The most remarkable differences between α and β epimers are the upfield displacement in the chemical shift of H-1 (Δδ = −0.64 ppm) and the methylene protons on C-2 (Δδ = −0.14, 1.04 ppm) to β epimer. Fact that can be explained in terms of a change in the configuration of the C-10 hydroxyl group due to the A/B *trans* fusion for this compound. This fusion was confirmed by means of the ^13^C-NMR spectrum (Table 2), in which the chemical shift of the carbons adjacent to the A/B ring fusion (C-1, C-2, C-6 and C-16) are displaced upfield by 3.3, 1.2, 5.3 and 5.2 ppm respectively. On the other hand, C-5 and C-18 are displaced downfield by 2.5 and 5.9 ppm, in comparison to **1**, due to the decompressing effect of the *trans* A/B fusion.

**Table 1:** ^1^H-NMR data for compounds **1** and **2**. (300 MHz, TMS, *J *= Hz) ^a^.

**H**	**1** (δ ppm) ^b^	**1** (δ ppm) ^c^	**1 NOESY**	**2** (δ ppm) ^c^	**2 NOESY**
1	4.18 (1H)	3.97 (1H)	H-2α, H-11α,	3.33 (1H)	2α, H-19 pro-*S*
	dd, *J *= 9.3, 1.8	dd, *J *= 9.9, 2.7	H-11β	dd, *J *= 12.3, 5.7	
2α_eq_	2.97 (1H)	2.93 (1H)	H-1, H-2β, H-3	2.71 (1H)	H-1α, H-2β,
	ddd *J *= 21.0, 9.6, 4.2	ddd, *J *= 21.0, 9.9, 4.8		dt, *J *= 20.7, 5.7	H-3
2β_ax_	3.28 (1H)	3.42 (1H)	H-2α, H-3	2.38 (1H)	H-2α, H-3
	ddd, *J *= 21.0, 3.6, 2.1	dt, *J *= 21.0, 3.0		ddd, *J *= 20.7, 12.3, 3.0	
3	6.93 (1H) dd, *J *= 4.2,	7.09 (1H)	H-2α, H-2β	7.18 (1H)	H-2α, H-2β
	3.6	t, *J *= 3.6		dd, *J *= 5.7, 3.0	
6α_eq_	1.97 (1H)	2.08 (1H)	H-6β, H-7,	1.86 (1H)	H-6β, H-7
	m	dd, *J *= 15.6, 3.0	H-19 pro-*R*	dd, *J *= 15.6, 2.4	
6β_ax_	1.06 (1H)	1.10 (1H)	H-6α, H-7, H-8	1.79 (1H)	H-6α, H-7, H-8
	ddd,*J *= 15.6, 3.6, 2.1	dt, *J *= 15.6, 3.0, 2.1		ddd, *J *= 15. 6, 4.5, 1.5	
7	4.83 (1H)	4.9 (1H)	H-6α, H-6β,	5.12 (1H)	H-6α, H-6β,
	q, *J *= 3.9	dt, *J *= 6.5, 3.3	H-8, Me-17	td, *J *= 4.2, 2.4	H-8, Me-17
8	1.97 (1H)m	1.95 (1H)qd, *J *= 7.0, 4.2	H-6β, H-7, Me-17	1.96 (1H)qd, *J *= 7.2, 4.2	H-6β, H-7,Me-17
11α	2.48 (1H)	2.62 (1H)	H-1α, H-11β,	2.48 (1H)	H-11β, Me-17,
	d, *J *= 17.1	d, *J *= 17.7	Me-17, Me-20	d, *J *= 15.6	Me-20
11β	3.43 (1H)	3.3 (1H)	H-1α, H-11α,	3.52 (1H)	H-11α
	d, *J *= 17.1	d, *J *= 17.7		d, *J *= 15.6	
14	6.63 (1H)	6.72 (1H)	H-15	6.71 (1H)	H-15
	d, *J *= 1.4	d, *J *= 1.8		d, *J *= 2.0	
15	7.57 (1H)	7.35 (1H)	H-14	7.29 (1H)	H-14
	d, *J *= 1.4	d, *J *= 1.8		d, *J *= 2.0	
17	0.81 (1H)	0.83 (3H)	H-7, H-8,	0.95 (3H)	H-7, H-8,
	d, *J *= 6.9	d, *J *= 7.0	H-11α, Μe-20	d, *J *= 7.2	H-11α, Μe-20
19 pro-*R*	4.93 (1H)	5.0 (1H)	H-6α, H-19 pro-*S*,	4.7 (1H)	H-19 pro-*S*,
	d, *J *= 8.1	d, *J *= 8.7	Me-20	d, *J *= 8.4	Me-20
19 pro-*S*	4.50 (1H)	4.47, (1H)	H-19 pro-*R*,	4.52, (1H)	H-1, H-19 pro-*R*
	dd, *J *= 8.1, 2.1	dd, *J *= 8.7, 2.1	Me-20	dd, *J *= 8.4, 1.5	
20	1.15 (3H)	1.15 (3H)	H-11α, Me-17, H-19	1.23 (3H)	H-11α, Me-17,
	s	s	pro-*S* and pro-*R*	s	H-19 pro-*R*
CH_3_COO		2.10 (3H)		2.10 (3H)	
		s		s	
-OH		2.2		3.38	

^a^ Assignments confirmed by COSY, HETCOR and NOESY NMR experiments; ^b^ Acetone-*d*_6_ used as solvent; ^c^ CDCl_3_ used as solvent.

**Table 2:** ^13^C-NMR data for compounds **1** and **2**. (75 MHz, CDCl_3_, TMS) ^a^.

**C**	**1** (δ ppm)	**2** (δ ppm)
1	42.7 d	39.4 d
2	23.6 t	22.4 t
3	133. 6d	136.1 d
4	130.8 s	132.5 s
5	46.1 s	48.6 s
6	34.7 t	29.4 t
7	70.9 d	72.0 d
8	37.2 d	37.3 d
9	45.3 s	46.4 s
10	77.6 s	77.8 s
11	52.0 t	52.2 t
12	194.6 s	194.2 s
13	125.7 s	122.1 s
14	109.1 d	110.1 d
15	142.4 d	142.4 d
16	159.8 s	154.6 s
17	13.0 q	11.6 q
18	169.7 s	175.7 s
19	72.5 t	73.2 t
20	20.0 q	21.4 q
CH_3_COO	21.2 t	21.2 q
CH_3_ COO	169.0 s	169.9 s

^a^ Assignements confirmed by HETCOR and FLOCK experiments.

Although long range coupling between H-19 *pro S* and H-6β_ax_ was observed in **1** and **2**; H-6β_ax_ in **2** was displaced downfield 0.69 ppm in comparison with the corresponding signal observed for the H-6β_ax_ proton in **1**. The structural disposition of **2** produces a deshielding on H-6β due to a 1–3 diaxial interaction with C-OH, a feature that is not observed in **1**. Me-17, H-8, and the AB system assigned to the C-11 methylene protons did not show any significant changes.

**Figure 2:**
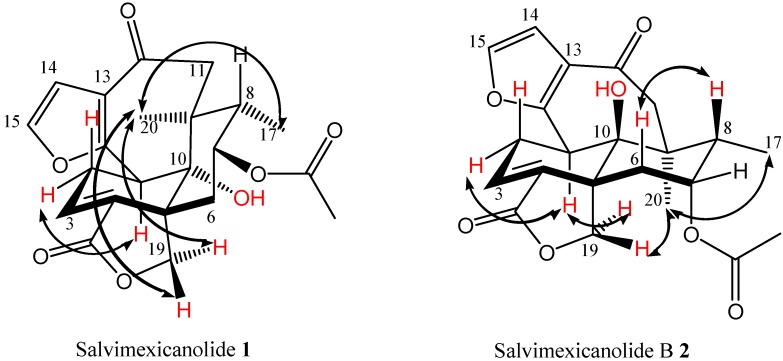
Key NOESY correlations.

The NOESY experiment confirmed the structures proposed for the epimers **1** and **2** (Figure 2). Thus, that experiment show the proton interaction between H-1-and C-11 methylene protons in **1**, which is not observed for the epimer **2**. The reason is that the epimer **1**, as can be observed in the Dreiding model, shows a boat conformation for the C ring due to the *cis* fusion of the A/B rings. According to the X-ray structure [7], this disposition induces an approach between the H-1 and the Hα-11 proton. Another remarkable difference observed for these products is that the NOESY spectrum for **2** showed the interaction between H-1 and H-19 pro-*S*, a fact supported by means of the analysis of the Dreiding model of this compound. This NOE interaction is due to the fact that the H-1 proton in the A/B *trans* languidulane skeleton is located close to H-19 pro-*S*. From a NOESY experiment performed on **1**, it was possible to assign the signals at δ 2.93 and 3.42 which correspond to H-2α and H-2β, whereas the same protons in **2** appear at δ 2.71 and 2.38, respectively (See Table 1).

It is noteworthy that the ^13^C-NMR data described in this paper shows important differences with the data previously reported for **1** [7], where C-1, C-6, C-13, C-16 were observed at higher chemical shift (3–5 ppm) and C-5, C-18 lower chemical shifts (2–6 ppm). We conclude that these differences are due to the fact that the reported ^13^C-NMR spectrum for salvimexicanolide (**1**) actually corresponds to the spectrum obtained for salvimexicanolide B (**2**) (see Table 2).

## 3. Experimental

### 3.1. General

Melting points were obtained with a Fisher-Johns apparatus and are uncorrected. EI-MS spectra were obtained at 70 eV by direct inlet injection on a Jeol JMS-AX505HA instrument. FAB HR-MS spectra were obtained with a Jeol JMS-SX102A. 1H-NMR (300 MHz) and 13C-NMR (75 MHz) spectra were recorded with a Varian Unity 300 spectrometer using TMS as internal standard. Infrared spectra were recorded in solution (CHCl_3_) using a Nicolet Magna 750 spectrophotometer. UV-spectra were recorded in a Shimadzu U-160 spectrophotometer. Optical rotatory measurements were performed in a Jasco DIP-360 polarimeter. 

Plant material was collected in April 1997, at 8 Km N from Izmolitla towards Eloxotitlan (Municipio Molango Hidalgo, México) and voucher specimens (MEXU 864121, 864122, 864123) were deposited in the Herbarium of the Instituto de Biología, UNAM México D.F.

### 3.1. Extraction and Isolation

Powdered, dried aerial parts of *S. mexicana* var*. mexicana* (1.2 kg) were extracted with Me_2_CO (3 × 10 L) at room temperature for one week. The solvent was removed under reduced pressure. During the evaporation of the solvent a mixture of ursolic and oleanolic acids was isolated by filtration (69 g). The extract (110 g) was partitioned using MeOH-H_2_O (4:1) and hexane-C_6_H_6_ (1:1). The polar fraction (32.2 g) was subjected to vacuum chromatography over silica gel. Mixtures of hexane-EtOAc of increasing polarity were used as eluents. Salvimexicanolide (**1**) precipitated from the fractions eluted with hexane-EtOAc (4:6). After exhaustive flash chromatography of these fractions, eluted with hexane-EtOAc (7:3), more salvimexicanolide (**1**) was obtained (0.074 g).

The fractions obtained from the vacuum chromatography eluted with hexane-EtOAc 7:3 to 1:1 (9.32 g) where subjected to a new vacuum chromatography separation over silica gel. Mixtures of hexane-EtOAc of increasing polarity were used as eluents. Dugesin B (**3**, 0.215 g) was obtained from the fractions eluted with hexane-EtOAc 6:4 to 1:1. Naringenin (**4**, 0.025 g) was isolated from fractions eluted with hexane-EtOAc 4:6. Both of them obtained after flash chromatography. From the last fractions eluted with hexane-EtOAc 4:6 and the first eluted with hexane-EtOAc 3:7, salvimexicanolide B (**2**) was obtained (0.045 g). The fractions (2.751 g) from the first vacuum chromatography, eluted with EtOAc and EtOAc-MeOH 9:1, were subjected to a new vacuum chromatography over silica gel. After the column was eluted with a mixture of EtOAc-MeOH 9.9:0.1, arbutin (**5**, 0.170 g) was isolated.

*Salvimexicanolide* (**1**). Rf (hexane-EtOAc 30:70): 0.6; Mp 265–267 °C; UV (MeOH), λ_max_ (ε): 274.5 (5940), 208 (21360); [α]_D_ + 233° (MeOH; *c* 0.1); IR (CHCl_3_) ν_max_ cm−1: 3581, 3112, 2941, 1755, 1715, 1661, 1429, 1369, 1126, 1033, 979; 1H-NMR see Table 1; 13C-NMR see Table 2; FAB-MS HR *m/z*: 401.1628 [M++1]; C_22_H_25_O_7_ requires [M++1] at *m/z* 401.1600. EI-MS *m/z* (rel. int.): 400 (100), 382 (17), 358 (55), 340 (26), 322 (10), 295 (27), 278 (12), 267 (26), 249 (24), 235 (23), 223 (22), 199 (17), 189 (19), 161 (23), 135 (41), 121 (38).

*Salvimexicanolide B* (**2**). Rf (hexane-EtOAc 30:70): 0.4; Mp 137–139 °C; UV (MeOH), λ_max_ (ε): 304.8 (12580), 218.8 (13620); [α]_D_ + 44° (MeOH; *c* 0.1); IR (CHCl_3_) ν_max_ cm−1: 3604, 2973, 2927, 1777, 1738, 1660, 1514, 1434, 1366, 1146, 1015; 1H-NMR see Table 1; 13C-NMR see Table 2; FAB-MS HR *m/z*: 401.1614 [M++1]; C_22_H_25_O_7_ requires [M++1] at *m/z* 401.1600. EI-MS *m/z* (rel. int.): 400 (70), 382 (9), 358 (100), 340 (35), 322 (7), 312 (13), 297 (8), 285 (8), 274 (12), 258 (26), 244 (23), 223 (12), 200 (52), 199 (34), 187 (15), 161 (13), 128 (13).

*Dugesin B* (**3**). Rf (hexane-EtOAc 30:70): 0.5; Mp 213–214 °C; UV (MeOH), λ_max_ (ε): 299 (9584), 239 (24500), 216 (33416); [α]_D_ − 120° (MeOH; *c* 0.1); IR (CHCl_3_) ν_max_ cm−1: 3110, 1759, 1604, 1296, 1080, 1026; 1H-NMR (CDCl_3_): 7.82 (s, 1H), 7.54 (d, *J* = 1.2, 1H), 7.40 (dd, *J* = 2.1, 1.2, 1H), 6.61 (d, *J* = 9.6, 1H), 6.46 (s, 1H), 6.09 (d, *J* = 2.1, 1H), 6.04 (dt, *J* = 9.6, 6.9, 1H), 5.47 (d, *J* = 17, 1H), 5.41 (d, *J* = 17, 1H), 3.22 (dd, *J* = 13.8, 6.9, 1H), 3.11 (dd, *J* = 13.8, 6.9, 1H), 2.29 (s, 3H). 13C-NMR (CDCl_3_): 172.4, 169.3, 155.3, 150.4, 144.3, 142.2, 141.7, 132.2, 130.9, 128.5, 126.7, 124.3, 122.1, 120.7, 119.8, 108.7, 75.1, 69.8, 29.8, 15.6; FAB-MS HR *m/z*: 335.0943 [M++1]; C_20_H_15_O_5_ requires [M++1] at *m/z* 335.0919. EI-MS *m/z* (rel. int.): 334 (100), 319 (8), 305 (32), 277 (7), 239 (71), 210 (6), 202 (4), 189 (5), 181 (5), 152 (6), 95 (10).

(-) *Naringenin* (**4**). Rf (hexane-EtOAc 30:70): 0.5; Mp 246–248 °C; IR (CHCl_3_) ν_max_ cm−1: 3290, 3120, 2918, 2833, 1634, 1601, 1517, 1464, 1311, 1251, 1179, 1156, 1084, 834; 1H-NMR 12.1 (s, 1-H, int-D_2_O, C_5_-OH), 9.6 (s, 1-H, int-D_2_O, C_7_-OH), 8.77 (s, 1-H, int-D_2_O, C_4_-OH), 7.41 (d, 2-H, *J* = 8.3, H-2’ H-6’), 6.92 (d, 2-H, *J* = 8.3, H-5’ H-3’), 5.96 (s, 2-H, H-6 H-8), 5.45 (dd, 1-H, *J* = 12.9, 3.1, H-2), 3.18 (dd, 1-H, *J* = 17.1, 12.9, H-3α), 2.72 (dd, 1-H, *J* = 17.1, 3.1, H-3β); 13C-NMR 197.3 (s, C-4), 167.3 (s, C-7), 165.2 (d, C-5), 164.3 (d, C-9), 158.6 (d, C-4’), 130.7 (s, C-1’), 128.9 (d, C-2’ C-6’), 116.1 (d, C3’ C-5’), 103.1 (s, C-10), 96.7 (d, C-6), 95.8 (d, C-8), 79.9 (d, C-2), 43.4 (t, C-3); EIMS *m/z* (rel. int.): 272 (100) [M+], 255 (7), 244 (5), 229 (5), 179 (22), 166 (23), 153 (71), 120 (41), 107 (12), 91 (14), 69 (13); C_15_H_12_O_5_ requires [M+] at *m/z* 272.

*Arbutin* (**5**). Rf (hexane-EtOAc-MeOH 30:60:10): 0.4; Mp 190–193 °C; IR (CHCl_3_) ν_max_ cm−1: 3335, 2910, 1649, 1512, 1444, 1402, 1215, 1103, 1066, 1012, 831, 615; 1H-NMR 8.14 (s, 1-H, int-D_2_O, Ph-OH), 6.92 (d, 2-H, *J* = 9, H-3’ H-5’), 6.78 (d, 2-H, *J* = 9, H-2’ H-6’), 4.47 (d, 2-H, *J* = 7.3, H-1), 4.66 (d, 1-H, *J* = 3.3, H-5), 4.4 (dd, 2-H, *J* = 16, 3.3, H-6), 3.95–3.60 (m, 3-H, H-2, H-3, H-4), 3.45 (s, 4-H, int.-D_2_O 4-OH); 13C-NMR 153.3, 151.9, 118.8, 116.3, 103.1, 77.9, 77.5, 74.6, 71.3, 62.61; EIMS *m/z* (rel. int.): 272 (1) [M+], 256 (1), 177 (2), 162 (5), 145 (3), 127 (3), 110 (100), 97 (4), 85 (6); C_12_H_16_O_7_ requires [M+] at *m/z* 272.

## 4. Conclusions

In summary, we were able to isolate ursolic and oleanolic acids, degustin B (**3**), the flavanone (-) naringenin (**4**), the substituted sugar arbutin (**5**) and two languidulanes, salvimexicanolide (**1**) and salvimexicanolide B (**2**) from the aerial part of *Salvia mexicana* var. *Mexicana.* Comparison of the spectra of isomers **1** and **2** with those earlier reported for the isomer **1**, indicated that the structure of salvimexicanolide (**1**) was not compatible with the ^13^C-NMR data described in the literature. The correct structure was assigned by detailed spectroscopic analysis of both isomers.

## Supplementary Materials

Supplementary materials can be accessed at: http://www.mdpi.com/1420-3049/16/10/8866/s1.

## Supplementary Materials

Supplementary File 1
