# Supplementary material for: A New Languidulane Diterpenoid from Salvia mexicana var. mexicana
Source: Molecules. 2011 Oct 21;16(10):8866–73. doi: 10.3390/molecules16108866 (PMC6264398; doi:10.3390/molecules16108866)
Supplement: Supplementary File 1 [file molecules-16-08866-s001.pdf]

Supporting Information

Correction of the Structure of Two Languidulane Diterpenoids from *Salvia Mexicana* var. *Mexicana*

Bernardo Antonio Frontana-Uribe, Martha V. Escárcega-Bobadilla, Rosa Estrada-Reyes, José Antonio Morales-Serna, Manuel Salmón, Jorge Cárdenas

Instituto de Química, Universidad Nacional Autónoma de México, Circuito Exterior, Ciudad Universitaria, 04510 México D. F.

Author to whom correspondence should be addressed; E-Mail: rjcp@unam.mx; Tel.: +52-55-5622-4413; Fax: +52-55-5616-2217.

1. NMR Spectra for **4a** **S1**
2. NMR Spectra for **4b** **S7**

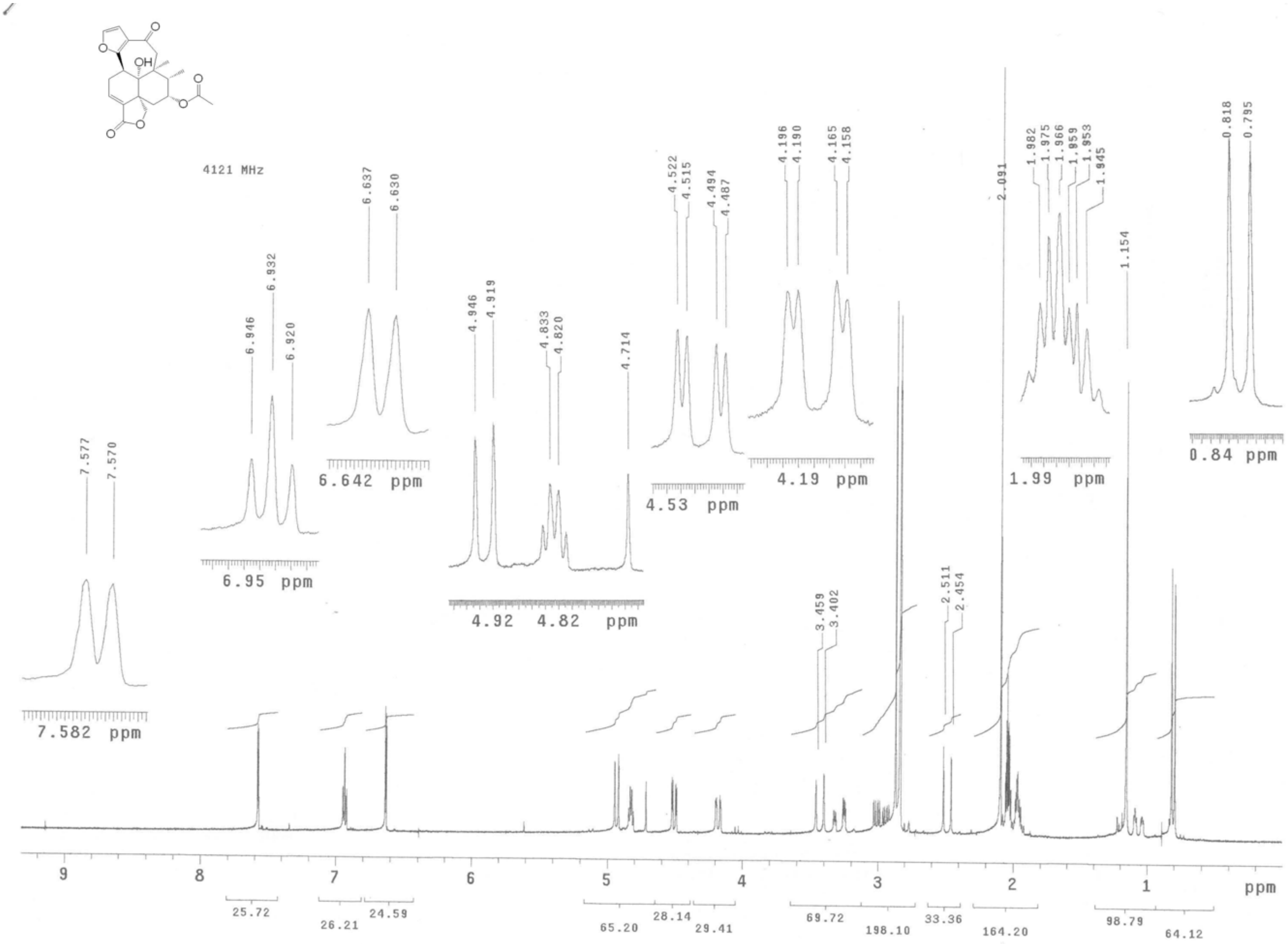

Figure S1. ^{1}H NMR of **4a** (acetone-d_6)

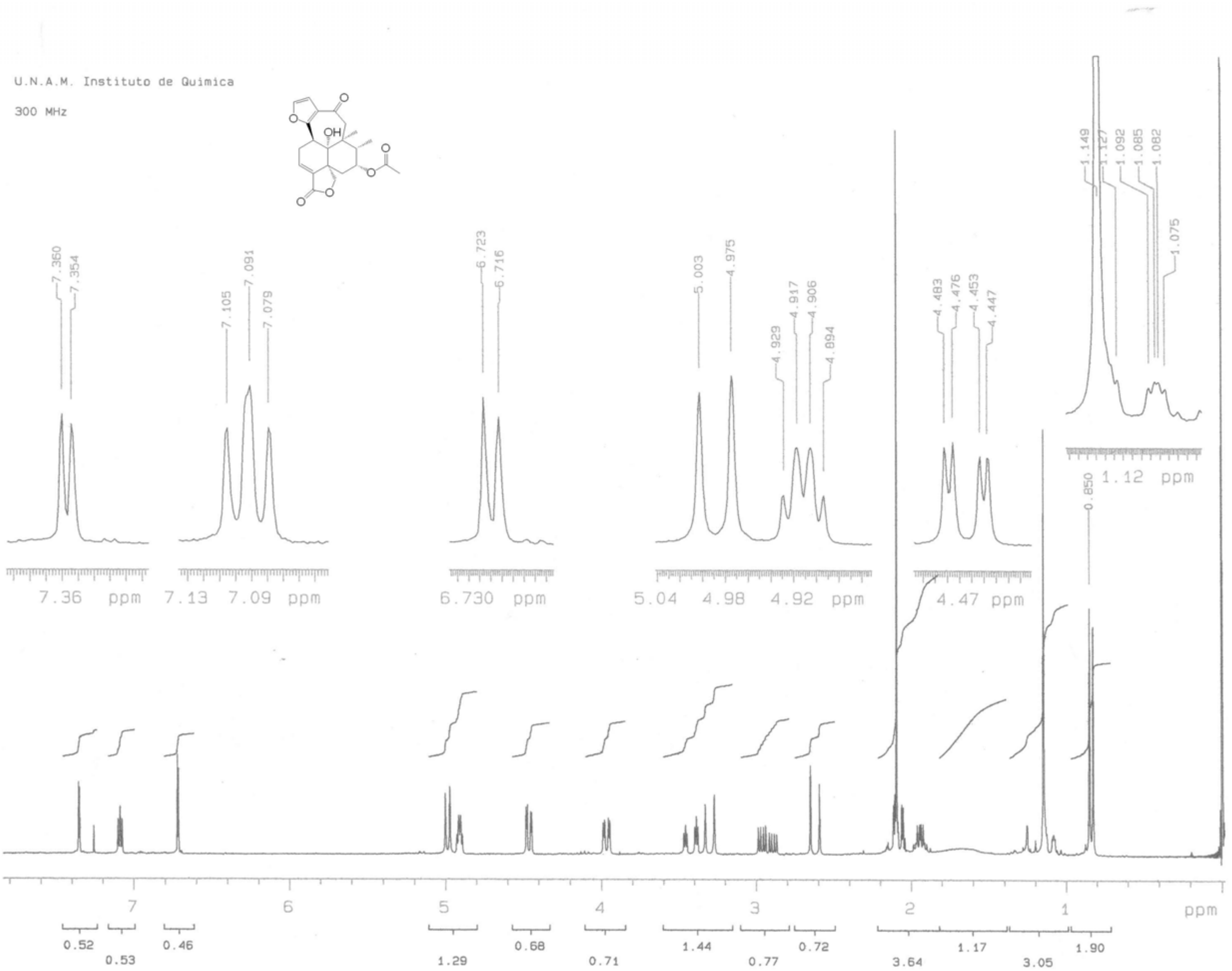

Figure S2. ^{1}H NMR of **4a** ($CDCl_3$)

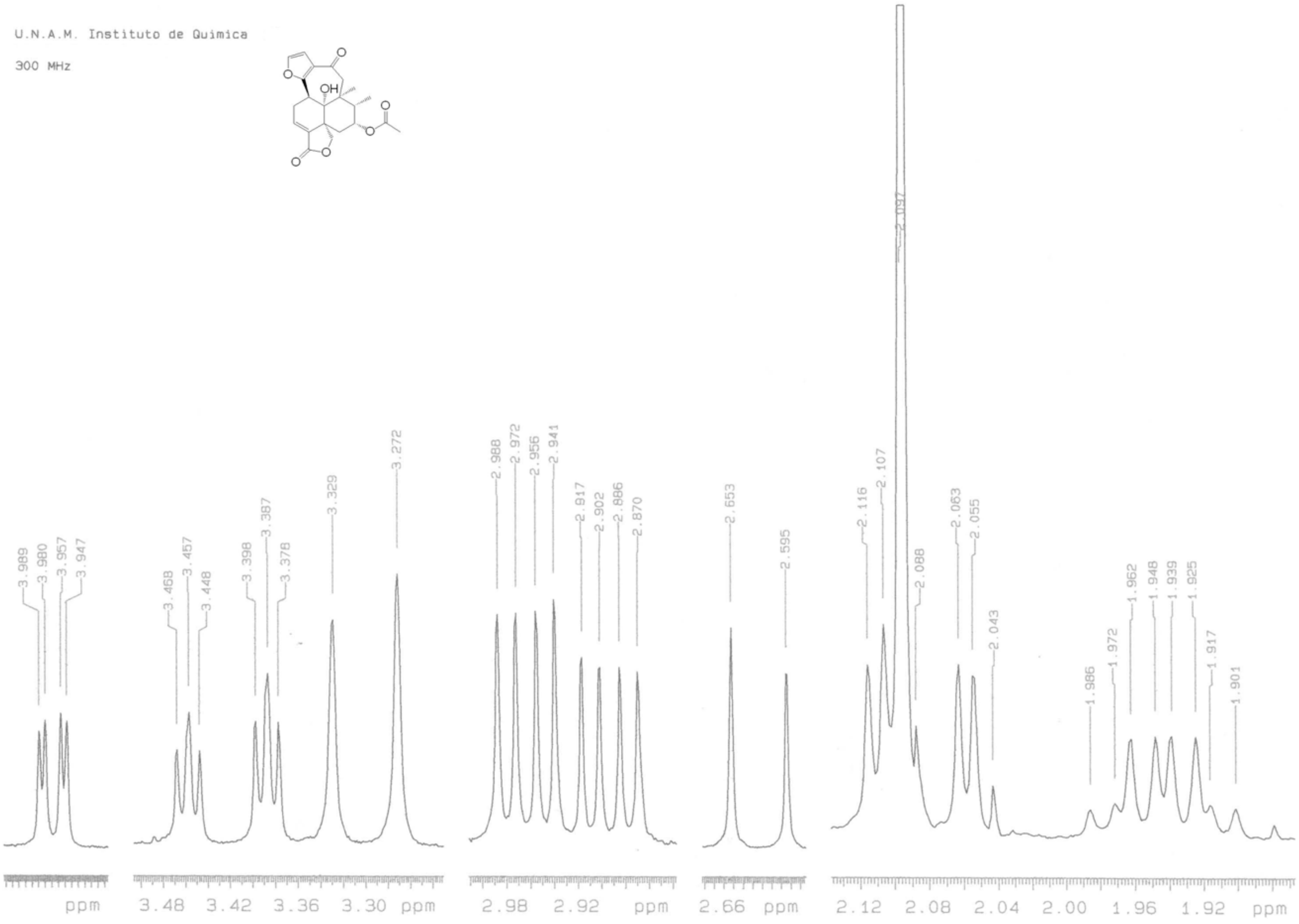

Figure S3. ^{1}H NMR of **4a** ($CDCl_3$)

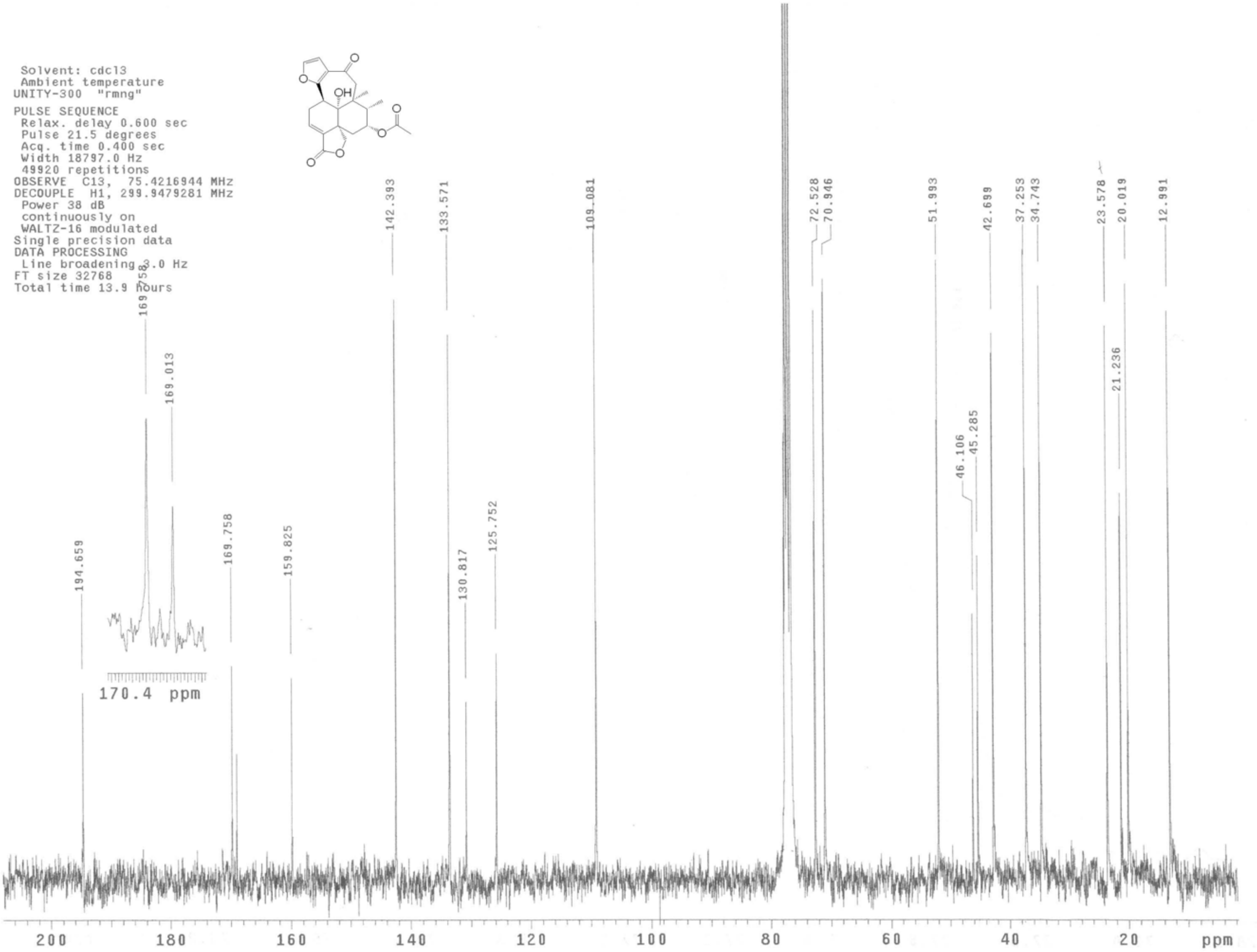

Figure S4. ^{13}C NMR of **4a** (CDCl$_3$)

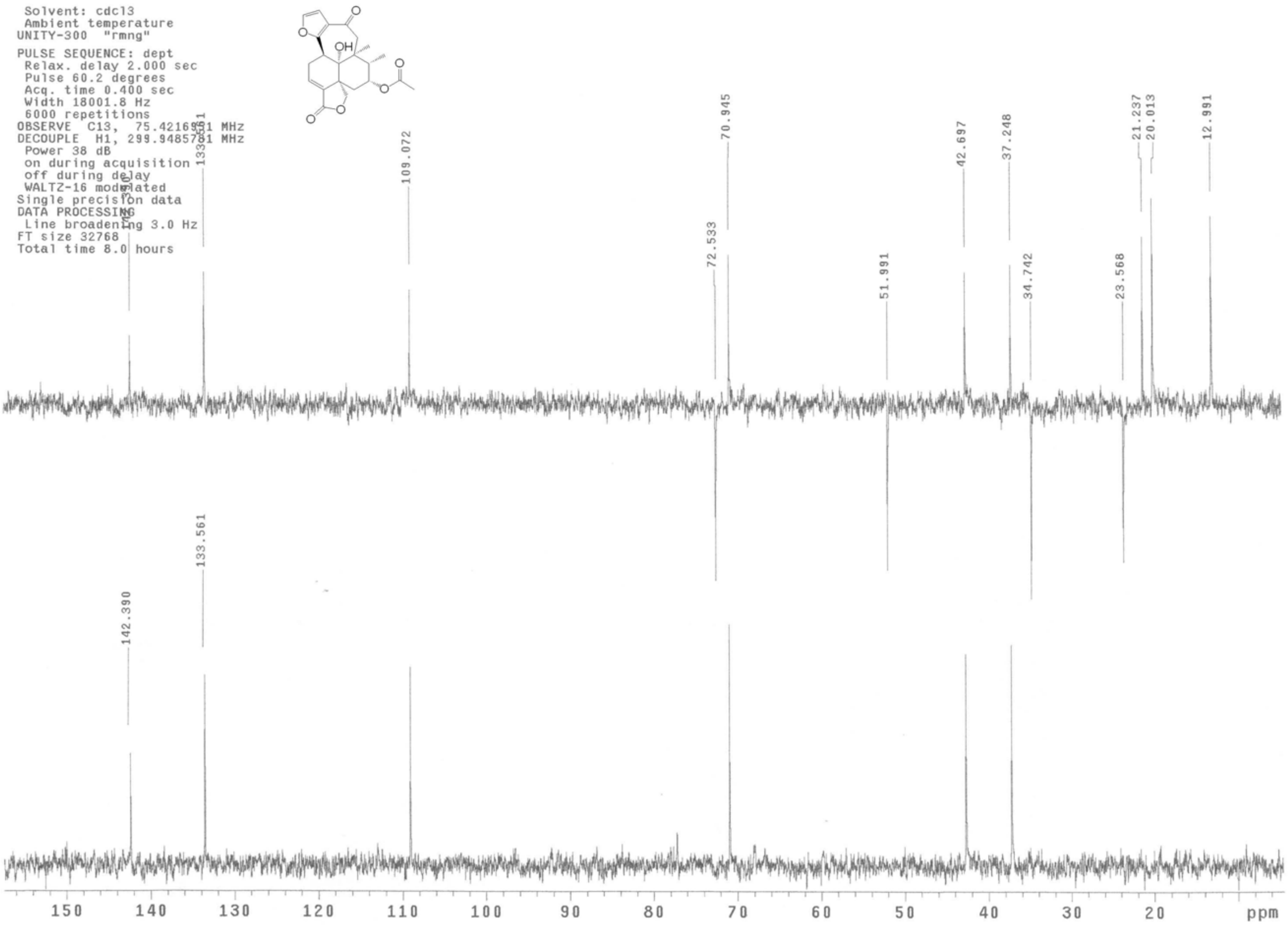

Figure S5. ^{13}C NMR DEPT of **4a** ($CDCl_3$)

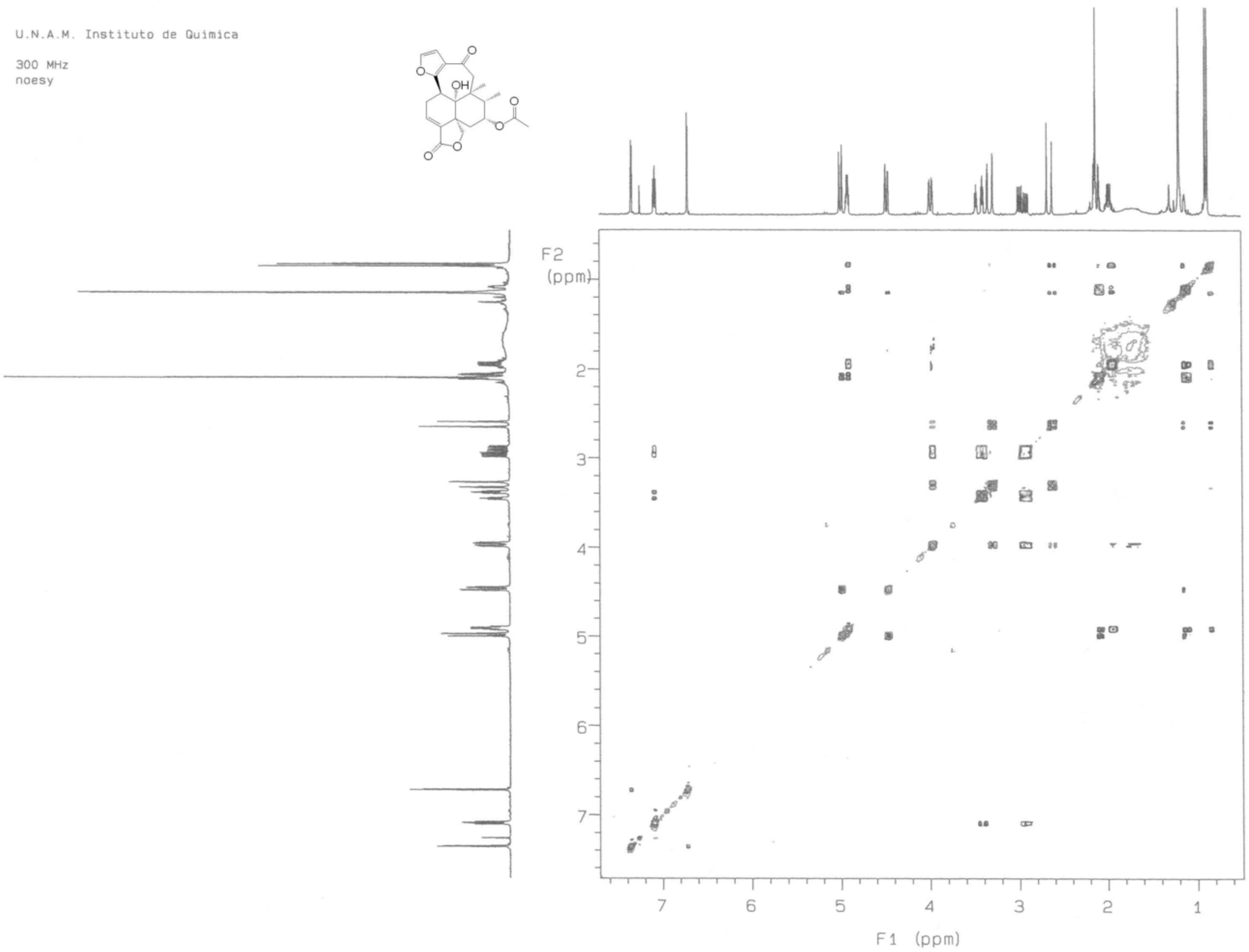

Figure S6. ^{1}H NMR COSY of **4a** ($CDCl_3$)

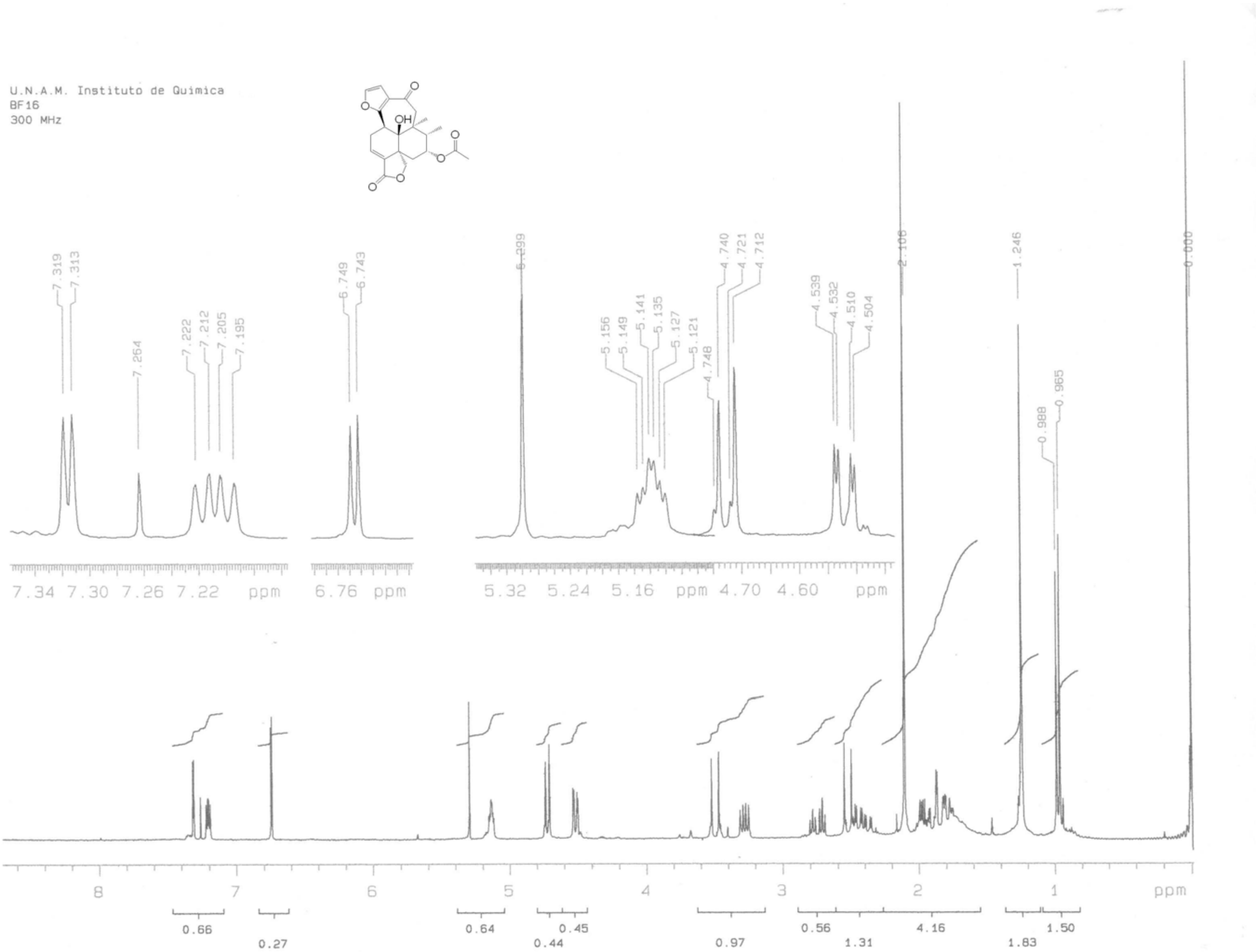

Figure S7. ^{1}H NMR of **4b** ($CDCl_3$)

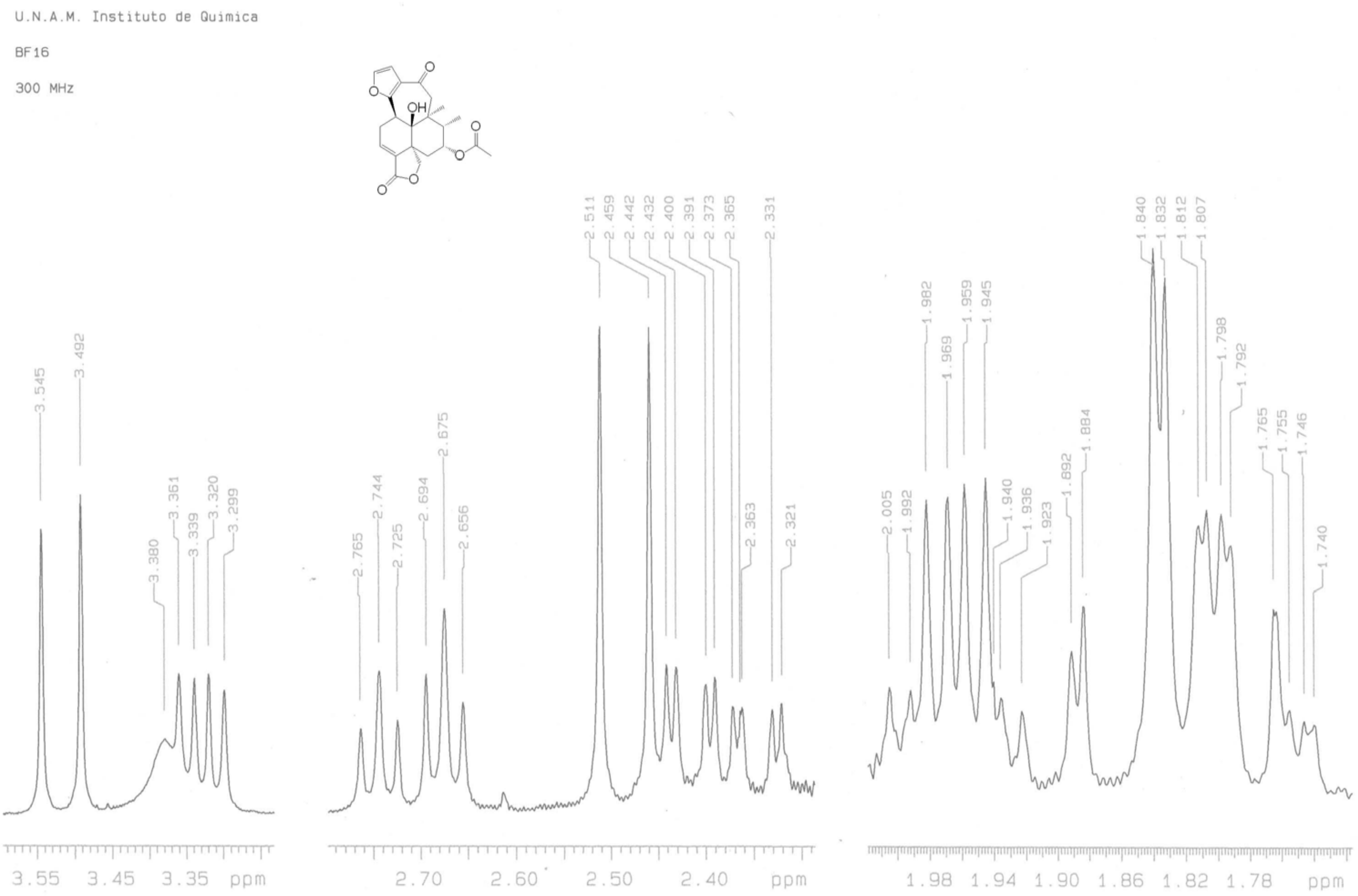

Figure S8. ^{1}H NMR of **4b** ($CDCl_3$)

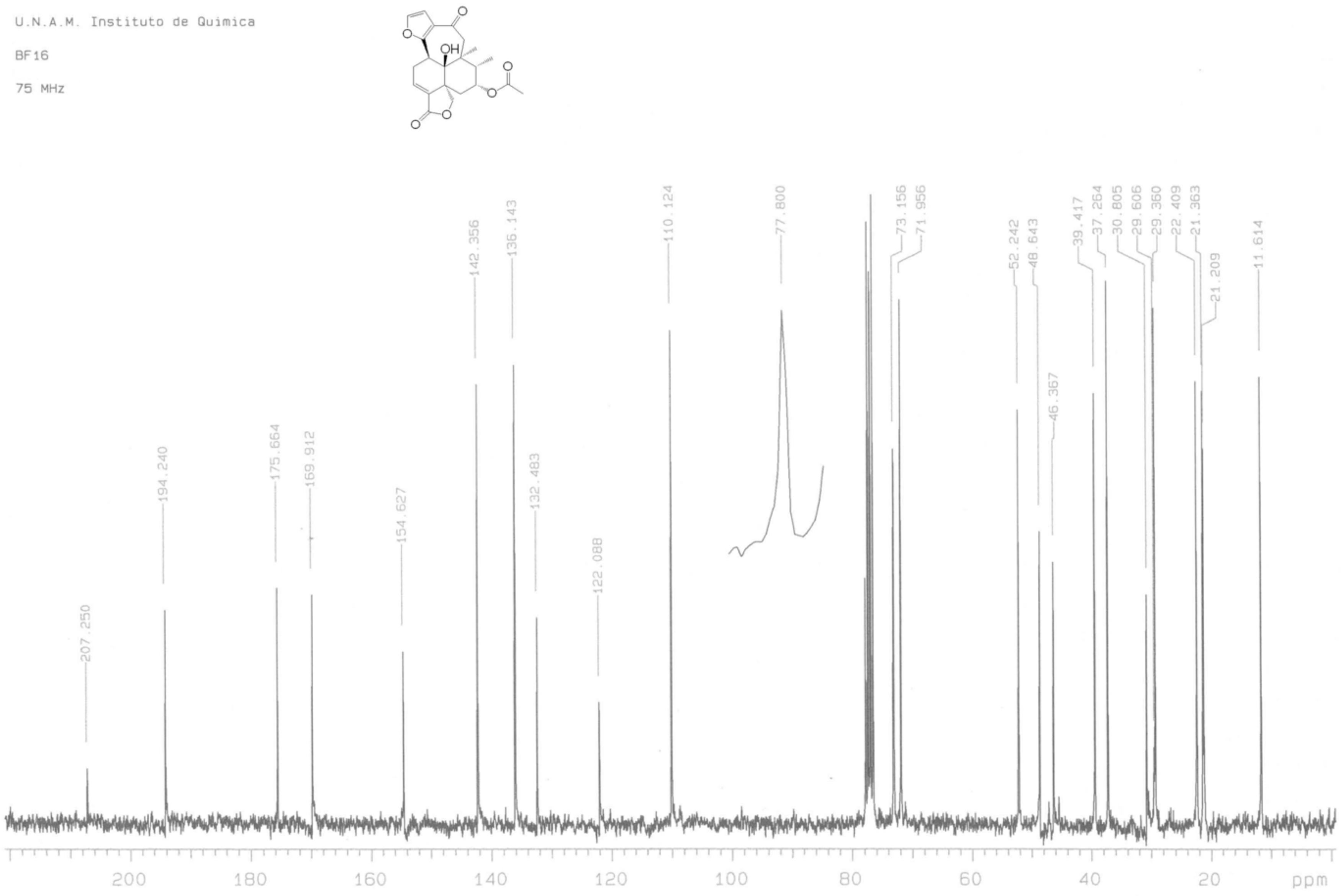

Figure S9. ^{13}C NMR of **4b** ($CDCl_3$)

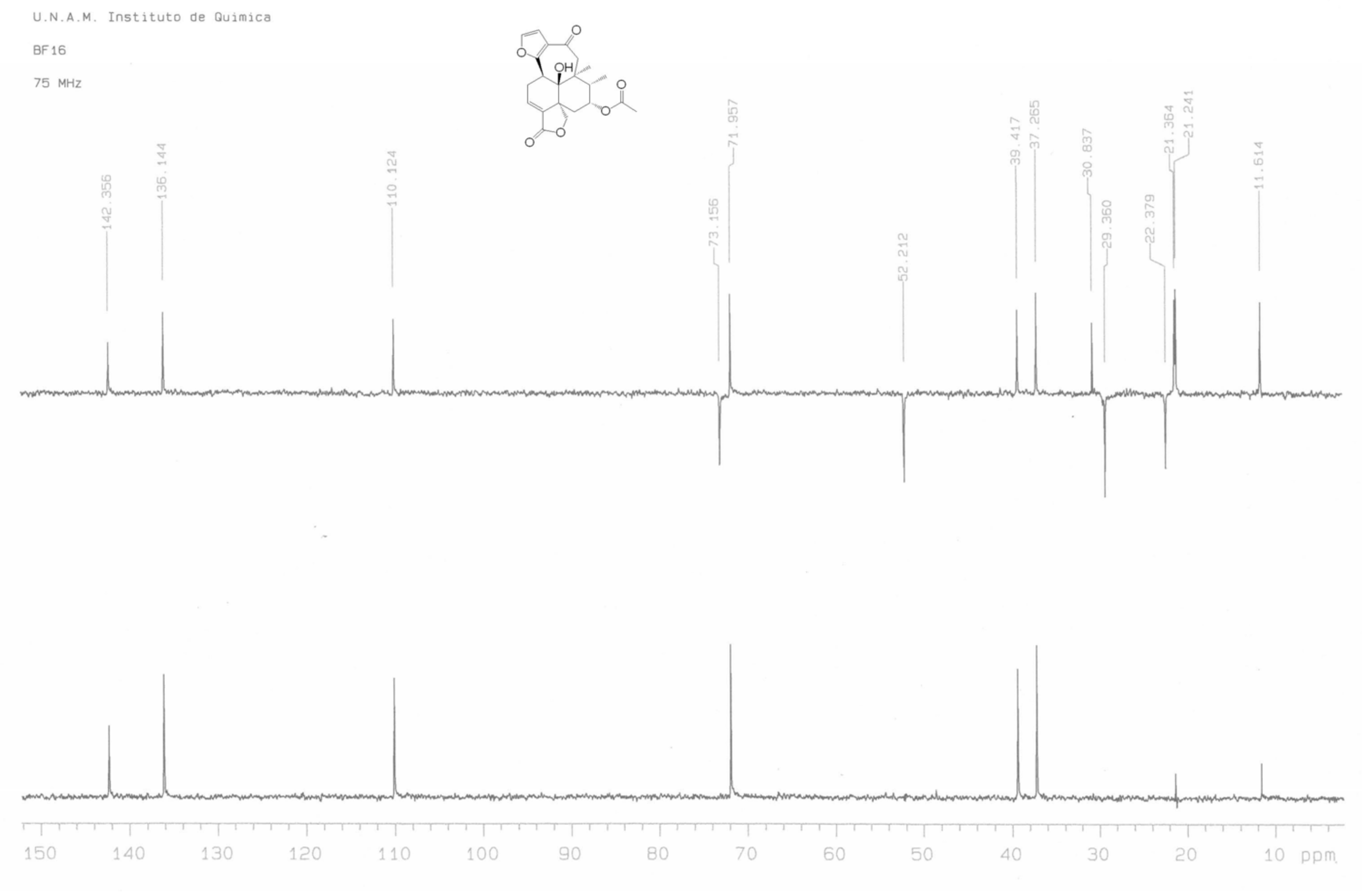

Figure S10. ^{13}C NMR DEPT of **4b** ($CDCl_3$)

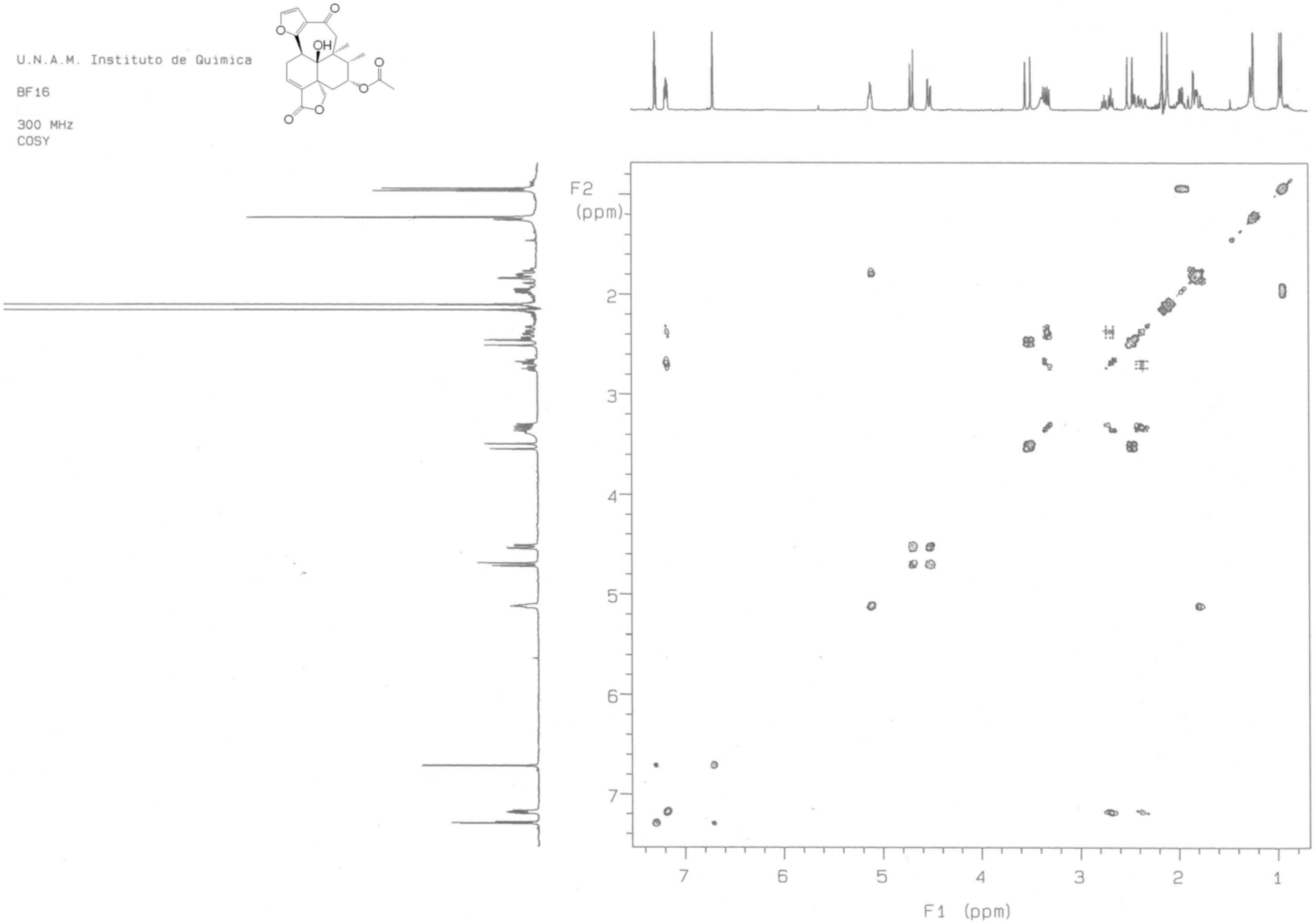

Figure S11. ^{1}H NMR COSY of **4b** ($CDCl_3$)

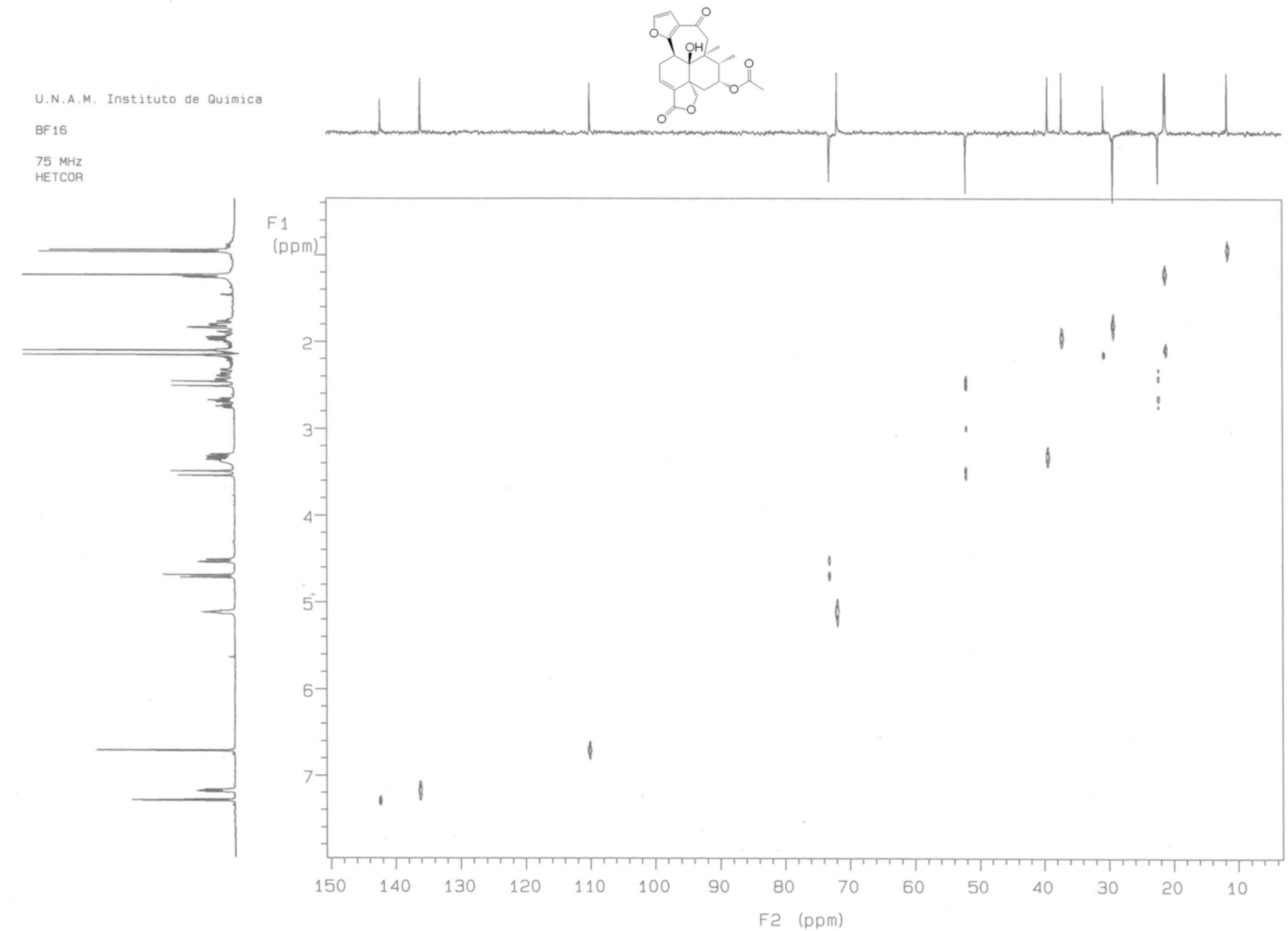

Figure S12. NMR HETCOR of **4a** ($CDCl_3$)

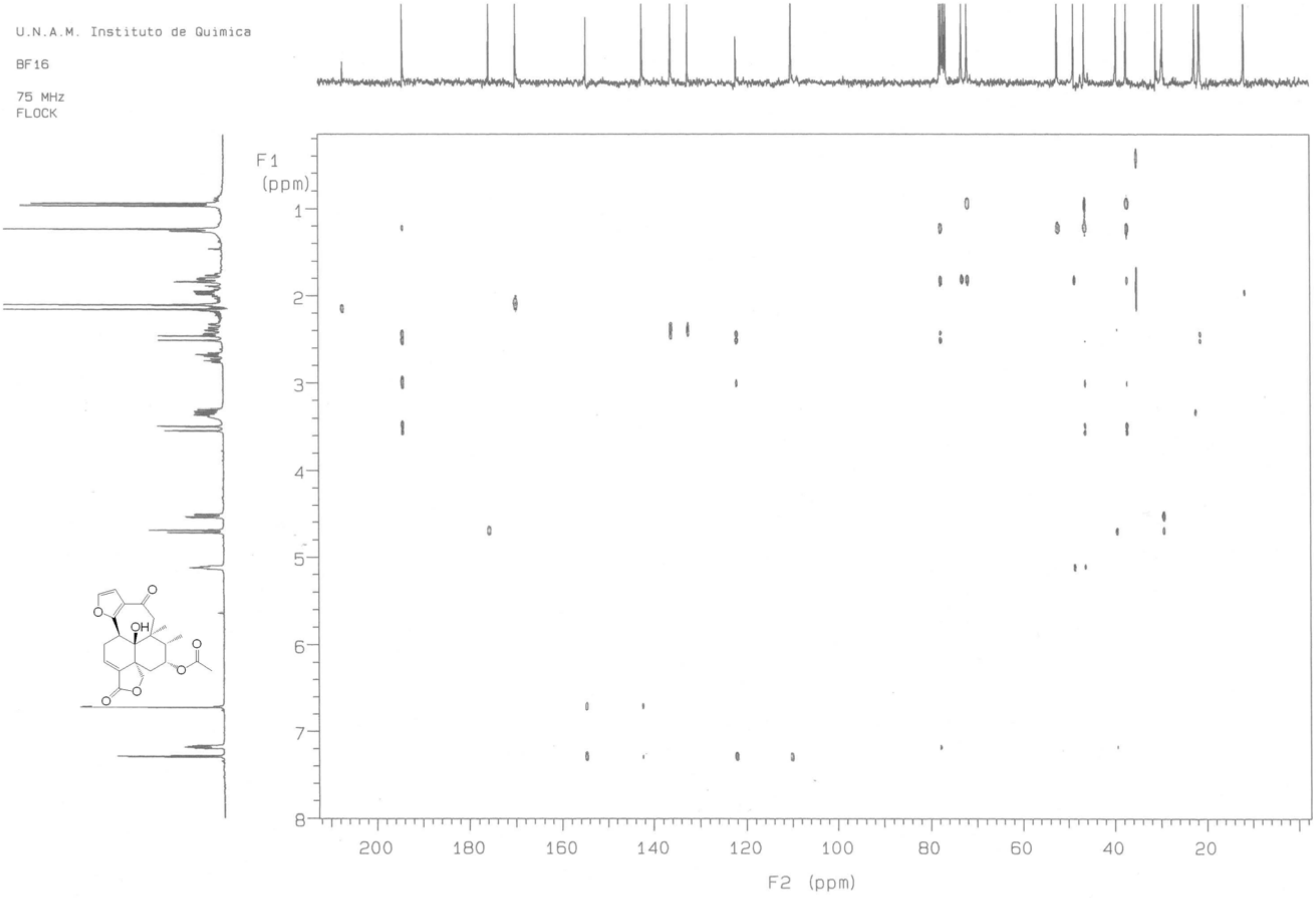

Figure S13. NMR FLOCK of **4b** ($CDCl_3$)

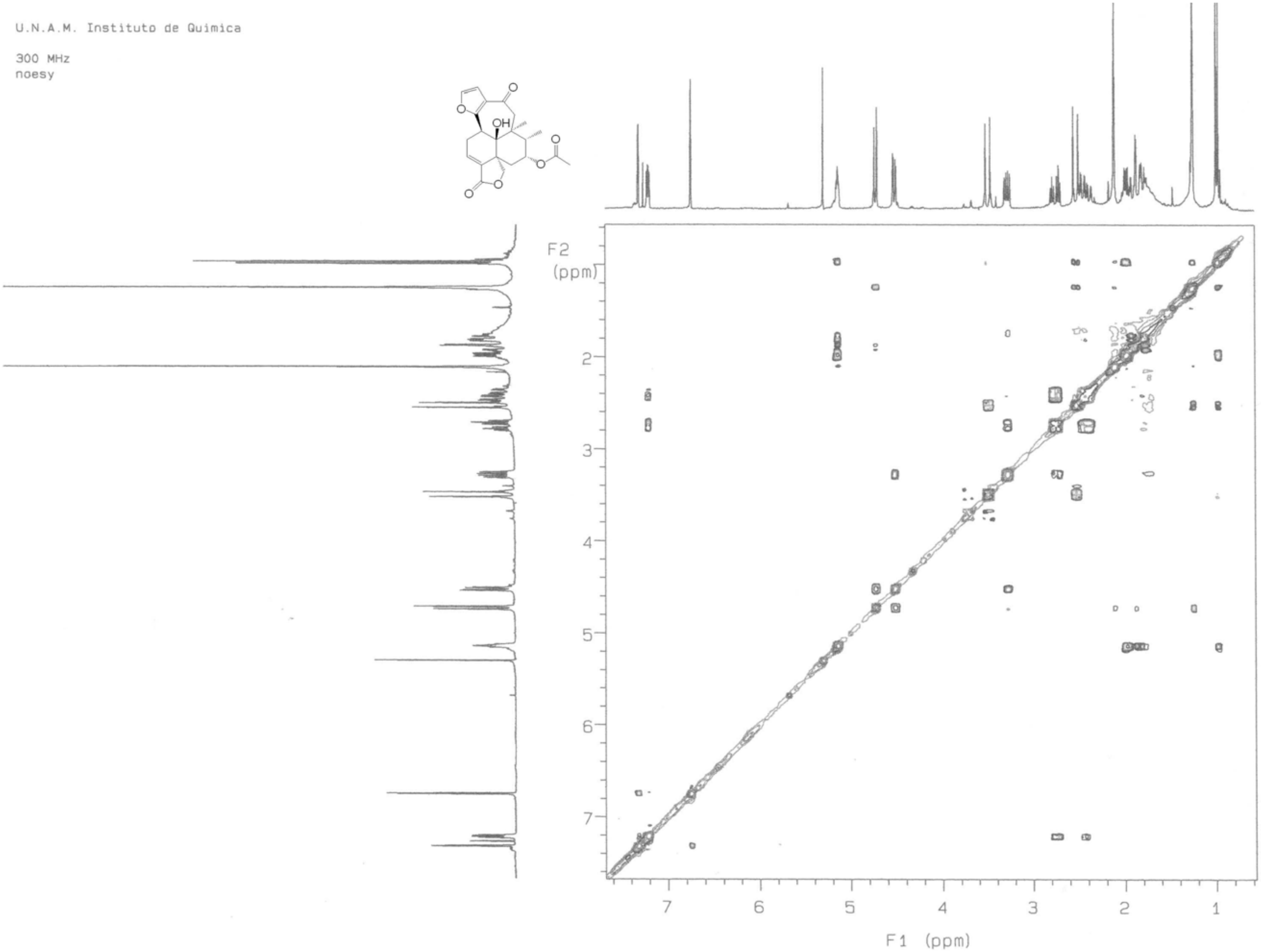

Figure S14. ^{1}H NMR NOESY of **4b** ($CDCl_3$)
